# Vertical distraction osteogenesis of a reconstructed mandible with a free vascularized fibula flap: a report of two cases

**DOI:** 10.1186/s40902-018-0172-2

**Published:** 2018-11-15

**Authors:** Naoaki Saito, Akinori Funayama, Yoshiaki Arai, Daisuke Suda, Yoshiyuki Takata, Tadaharu Kobayashi

**Affiliations:** 1 Division of Reconstructive Surgery for Oral and Maxillofacial Region, Department of Tissue Regeneration and Reconstruction, Course for Oral Life Science, Niigata University Graduate School of Medical and Dental Sciences, 2-5274, Gakkocho-Dori, Chuo-Ku, Niigata, 951-8514 Japan; 2 Temporomandibular Joint Clinic, Niigata University Medical and Dental Hospital, 1-754, Asahimachi-Dori, Chuo-Ku, Niigata, 951-8520 Japan; 3 Department of Oral and Maxillofacial Surgery, Niigata City General Hospital, 463-7, Shumoku, Chuo-Ku, Niigata, 950-1197 Japan

**Keywords:** Vertical distraction osteogenesis, Segmental mandibular resection, Mandibular reconstruction, Free vascularized fibula flap, Dental implant

## Abstract

**Background:**

The free vascularized fibula flap presents many advantages such as sufficient length of the bony segment, good vascularization, better quality of the bone, and a long vascular pedicle, but it is also associated with some disadvantages with regard to prosthetic rehabilitation because of its limited height. Improvement in bone height is necessary for ideal dental implant treatment of reconstructed mandibles.

**Case presentation:**

For two squamous cell carcinoma patients, mandibular bone reconstruction was performed secondarily with the peroneal flap after tumor resection. Since the bone height was insufficient at the time of implant treatment, occlusion reconstruction by dental implant was performed after vertical distraction osteogenesis.

**Conclusions:**

Vertical distraction osteogenesis is a suitable treatment option for alveolar ridge deficiency resulting from fibula transplantation for mandibular reconstruction following tumor surgery.

## Background

Various methods and procedures using bone grafts and metal plates are applied in the reconstruction of mandibles after tumor resection. Particularly after the vascularized bone graft has been established [1], mandibles with a wide range of bone defects can be reconstructed, and improvements in masticatory function can also be obtained via the application of dental implants [2, 3].

The free vascularized fibula flap presents many advantages such as sufficient length of the bony segment, good vascularization, better quality of the bone, and a long vascular pedicle, but it is also associated with some disadvantages with regard to prosthetic rehabilitation with dental implants because of the height discrepancy between the native mandible and the transplanted fibula. However, distraction osteogenesis is a method used to increase the height of the bone by pulling and extending the bone itself in order to improve quantitative shortages of bone and substantial defects and has been applied to restore vertical mandibular deficiency [4, 5].

This report presents two cases of vertical distraction osteogenesis of a free revascularized fibula flap followed by implant therapy for mandibular reconstruction after segmental mandibulectomy.

## Case presentation

### Case 1

**Fig. 1:**
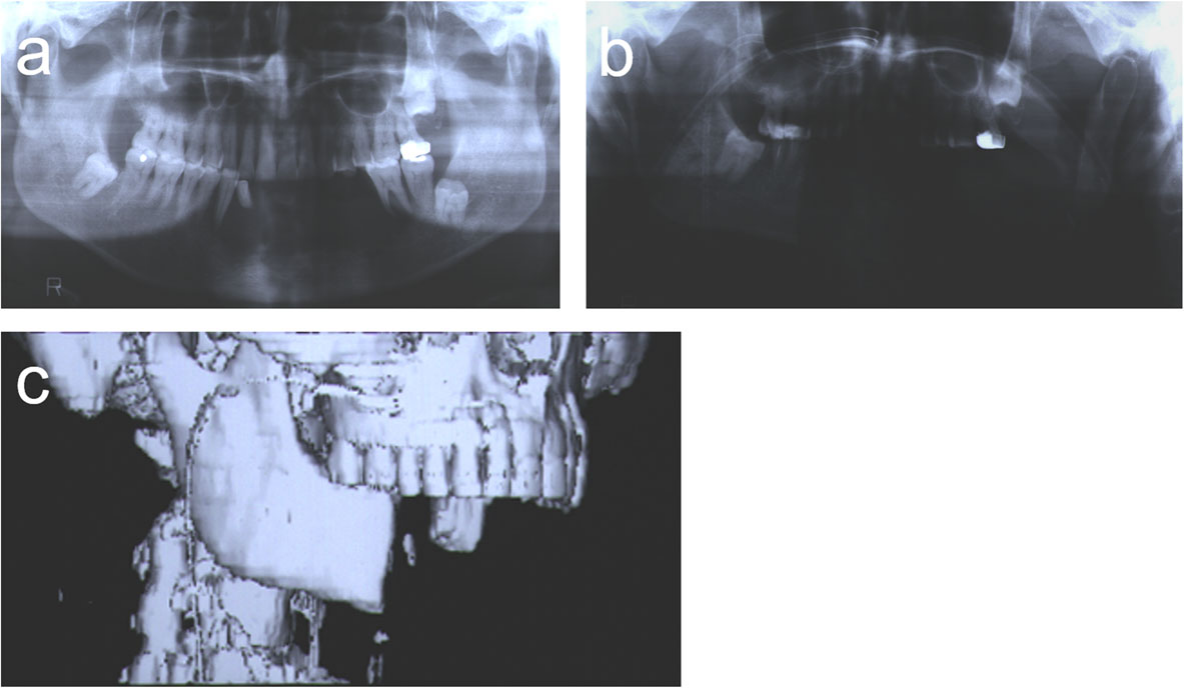
**a** Orthopantomogram before treatment. **b** Orthopantomogram and **c** 3-D computed tomography scan after segmental mandibular resection

**Fig. 2:**
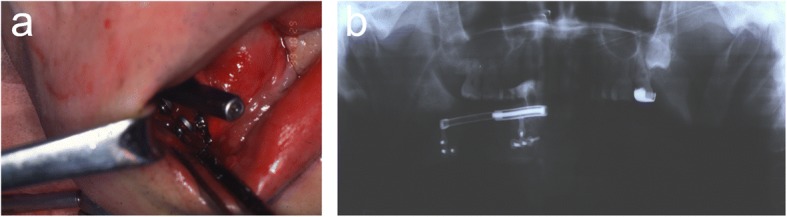
**a** Horizontal distraction osteogenesis of the remaining mandible was performed to reduce the bone defect. **b** Orthopantomogram after horizontal mandibular distraction

The patient was a 54-year-old man who was diagnosed with squamous cell carcinoma in the mandible on the right side. Under general anesthesia, segmental mandibular resection from the right first molar to the left ramus with bilateral radical neck dissection and perimandibular soft tissue resection were carried out. Immediate reconstruction of the soft tissue defect was performed with a forearm flap and a deltopectoral flap. Mandibular reconstruction was not performed primarily (Fig. 1). Horizontal distraction osteogenesis of the remaining mandible was performed 5 months later to reduce the bone defect (Fig. 2).

**Fig. 3:**
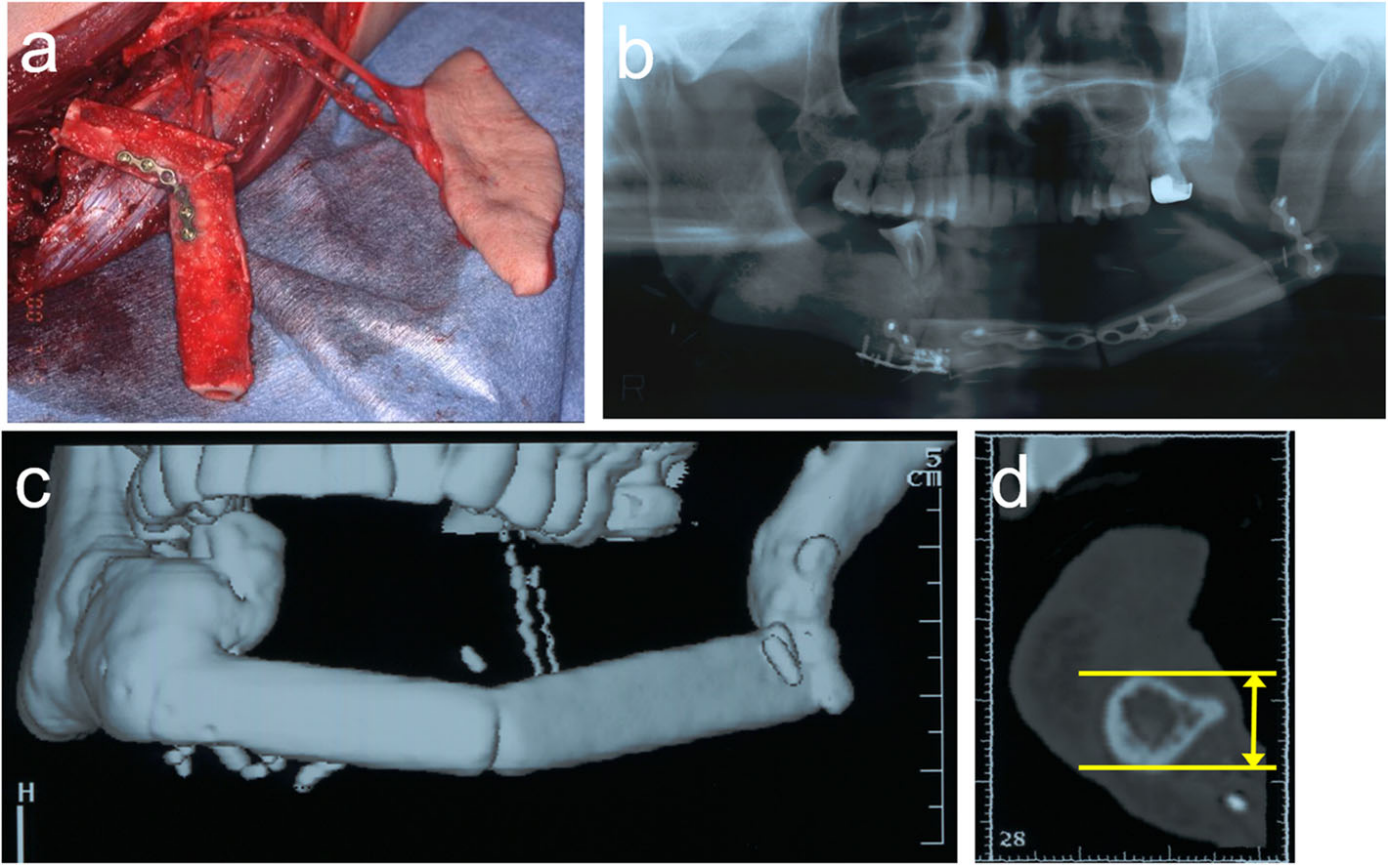
**a** A free vascularized fibula flap. **b** Orthopantomogram after mandibular reconstruction with a free vascularized fibula flap. **c** 3-D computed tomography scan and **d** cross sectional image 1 year after the mandibular reconstruction with a free vascularized fibula flap. The height of the fibula was 12 mm

The mandible was reconstructed secondarily using a free vascularized fibula flap 1 year after the first operation. The patient was then evaluated for implant therapy, but computed tomography (CT) and 3-D reconstruction images revealed that the height (12 mm) of the fibula was insufficient (Fig. 3).

**Fig. 4:**
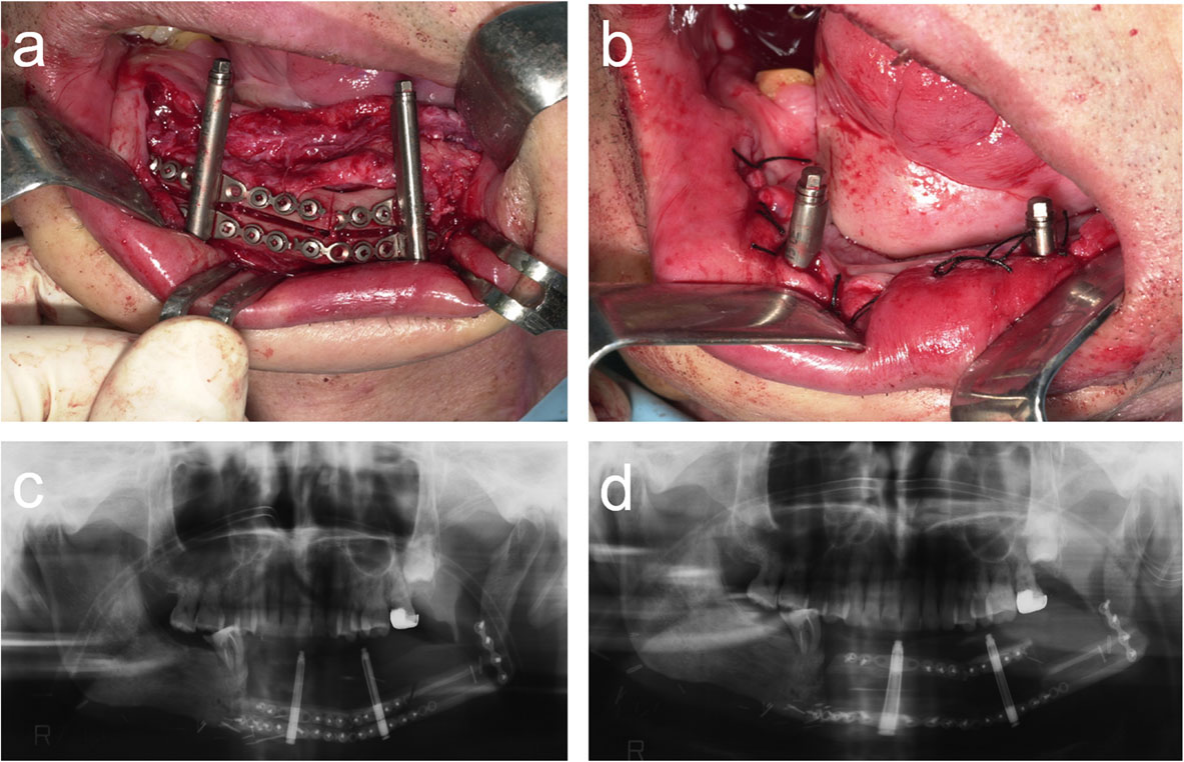
**a**, **b** Two distractors were fixed at the planned position. Orthopantomogram **c** 1 day postoperatively and **d** at the end of distraction osteogenesis

Vertical distraction osteogenesis of the fibula was performed under general anesthesia 1 year after the mandibular reconstruction. An intraoral incision in the buccal vestibule was performed, along with careful subperiosteal dissection to obtain adequate visibility of the underlying bone, taking care to preserve the lingual mucoperiosteal attachment. Two intraoral distraction devices (TRACK 1.5-mm system, KLS Martin L.P.) were adjusted and temporarily fixed by screws as planned before the osteotomy. After removal of the distractors, the osteotomy was performed with a sagittal saw on the vestibular aspect of the fibula. The distraction devices were fixed again at the planned position by screws and temporarily activated to a distance of approximately 5 mm to ensure correct function during distraction. Finally, the osteotomized segment was repositioned at its initial position, and the wound was sutured. After a 7-day latency period, the distraction devices were activated at a rate of 1 mm/day by turning the device twice a day for 13 days. The bone was distracted by approximately 13 mm (Fig. 4).

**Fig. 5:**
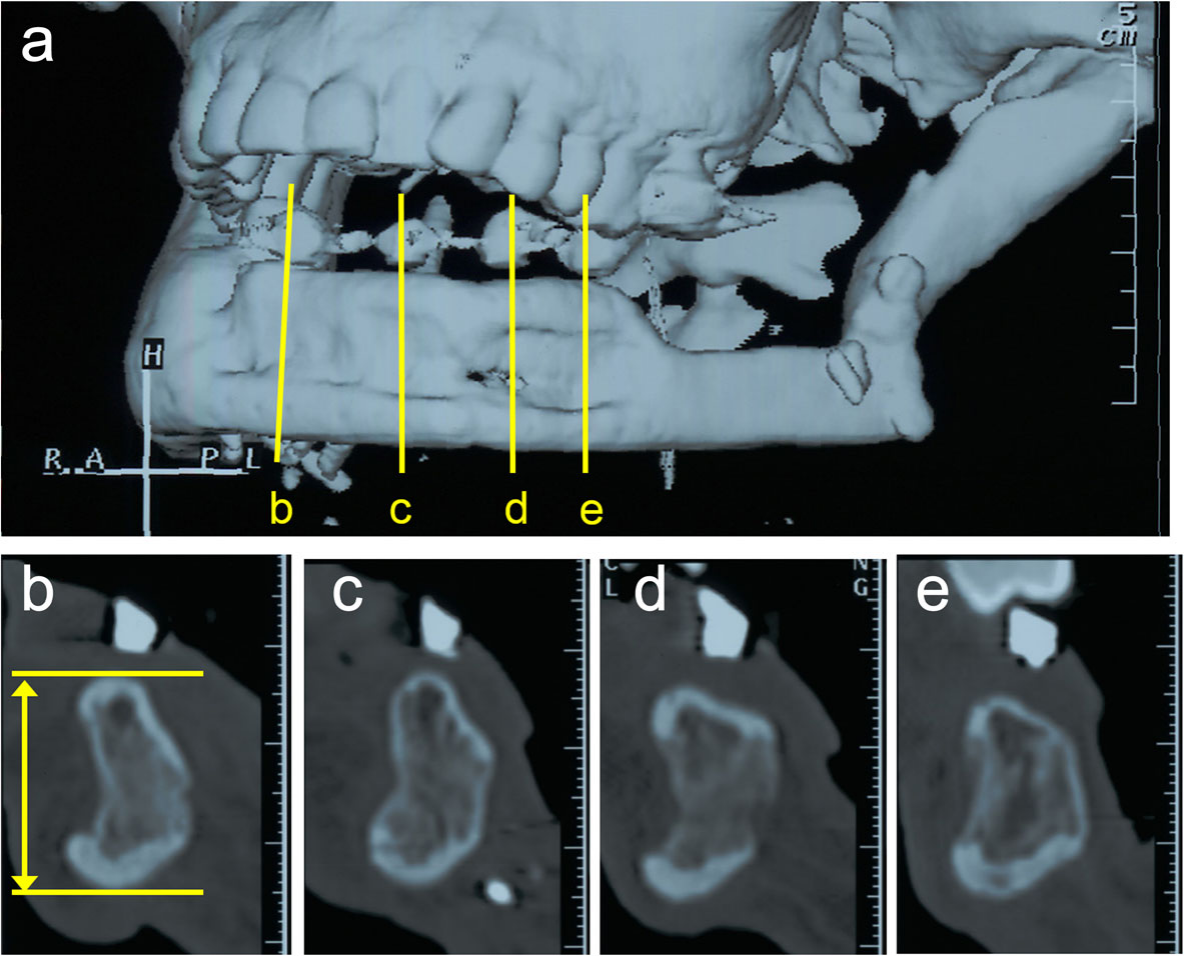
**a** 3-D computed tomography scan 7 months after vertical distraction osteogenesis of the free revascularized fibula flap. **b**–**e** Cross-sectional images of 7 months after vertical distraction. The height of the distracted fibula was approximately 23 mm

After a 4-month delay for bone consolidation, the distraction devices were removed, and good ossification was observed in the distracted area. The final bone height increase was 11 mm, as observed on CT and further demonstrated by 3-D reconstruction images, and the vertical discrepancy between the reconstructed mandible and the existing mandible was corrected (Fig. 5).

**Fig. 6:**
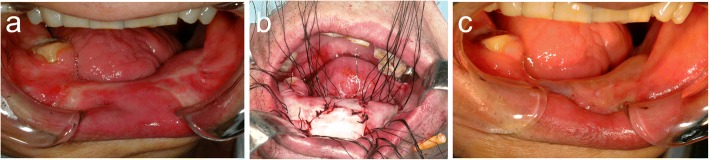
**a** Intraoral view 7 months after vertical distraction osteogenesis of the free revascularized fibula flap. **b** Vestibular extension of the free revascularized fibula flap using an ex-vivo produced oral mucosa equivalent. **c** Intraoral view 2 months after vestibular extension

After achieving the desired bone height, the vestibular extension was performed using a tissue-engineered oral mucosa (an ex vivo-produced oral mucosa equivalent: EVPOME) [6]. Autogenous keratinocytes were harvested from a punch biopsy 4 weeks prior to surgery, placed in a serum-free culture system, and seeded onto a human cadaveric dermal equivalent, namely AlloDerm. Clinically, the EVPOME grafts were easy to handle and exhibited excellent compliance with regard to grafting (Fig. 6).

**Fig. 7:**
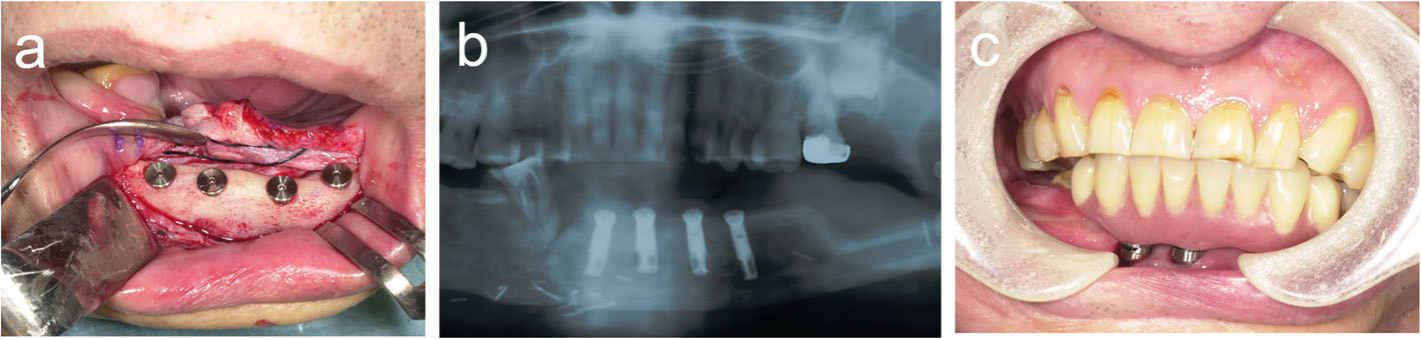
**a** Four dental implants were inserted into the distracted fibula. **b** Orthopantomogram after implantation. **c** Intraoral view after treatment: the implant denture was placed on the mandible

Four dental implants were inserted into the distracted fibula, and primary stability was achieved for all implants. Finally, the implant denture was placed on the mandible (Fig. 7).

### Case 2

**Fig. 8:**
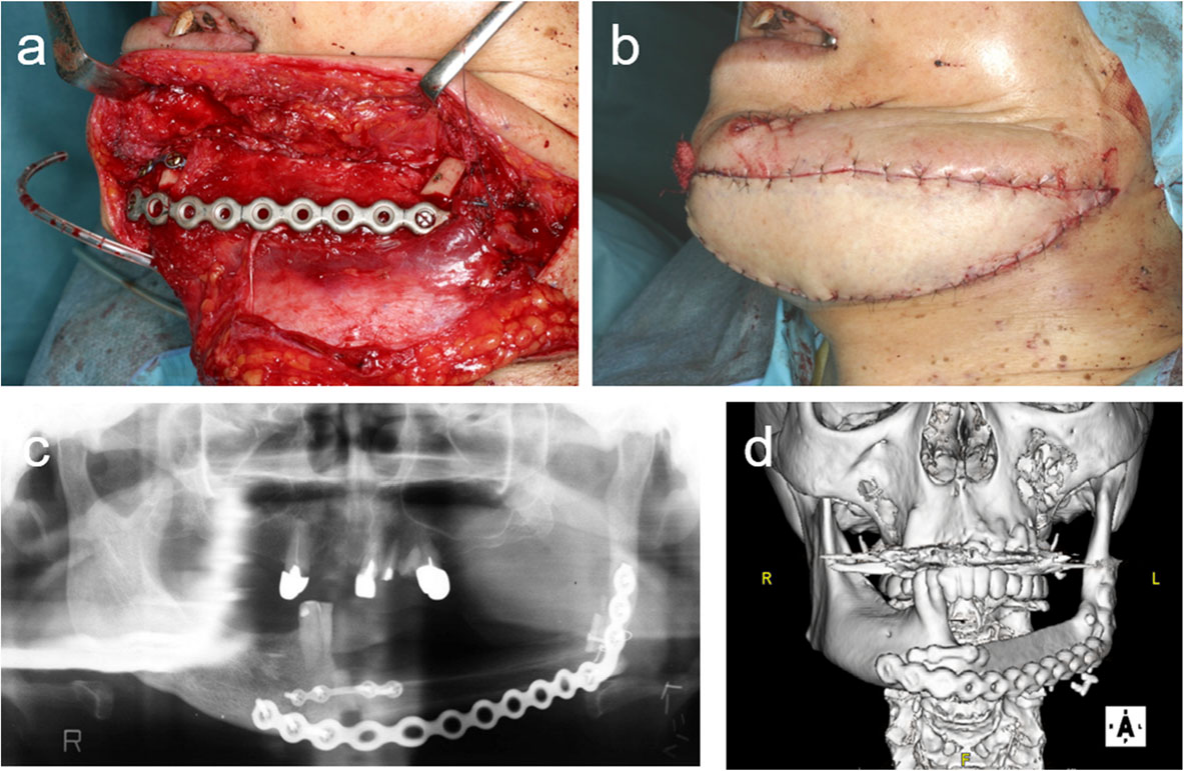
**a**, **b** The mandible was reconstructed secondarily with a free vascularized fibula flap 1 year and 7 months after segmental mandibular resection. **c** Orthopantomogram and **d** 3-D computed tomography scan after mandibular reconstruction with a free vascularized fibula flap

**Fig. 9:**
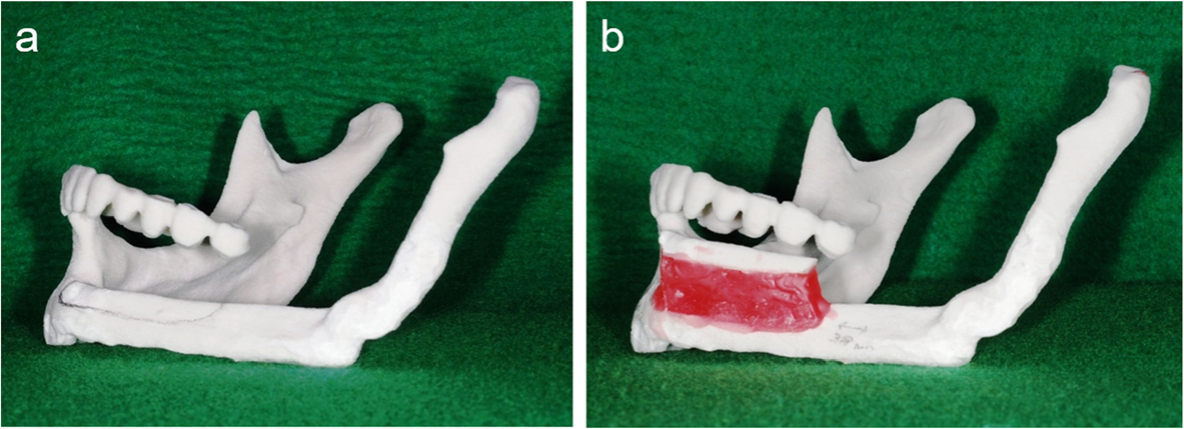
**a**, **b** Surgical simulation using a 3-D model

The patient was a 68-year-old female who had lower gingival squamous cell carcinoma in the left side of the mandible. Segmental mandibular resection from the right lateral incisor to the left ramus and resection with a titanium plate were carried out. The mandible was reconstructed secondarily with a free vascularized fibula flap 1 year and 7 months after the first operation (Fig. 8). CT and 3-D reconstruction images demonstrated the insufficient height (15 mm) of the fibula for implant therapy (Fig. 9).

**Fig. 10:**
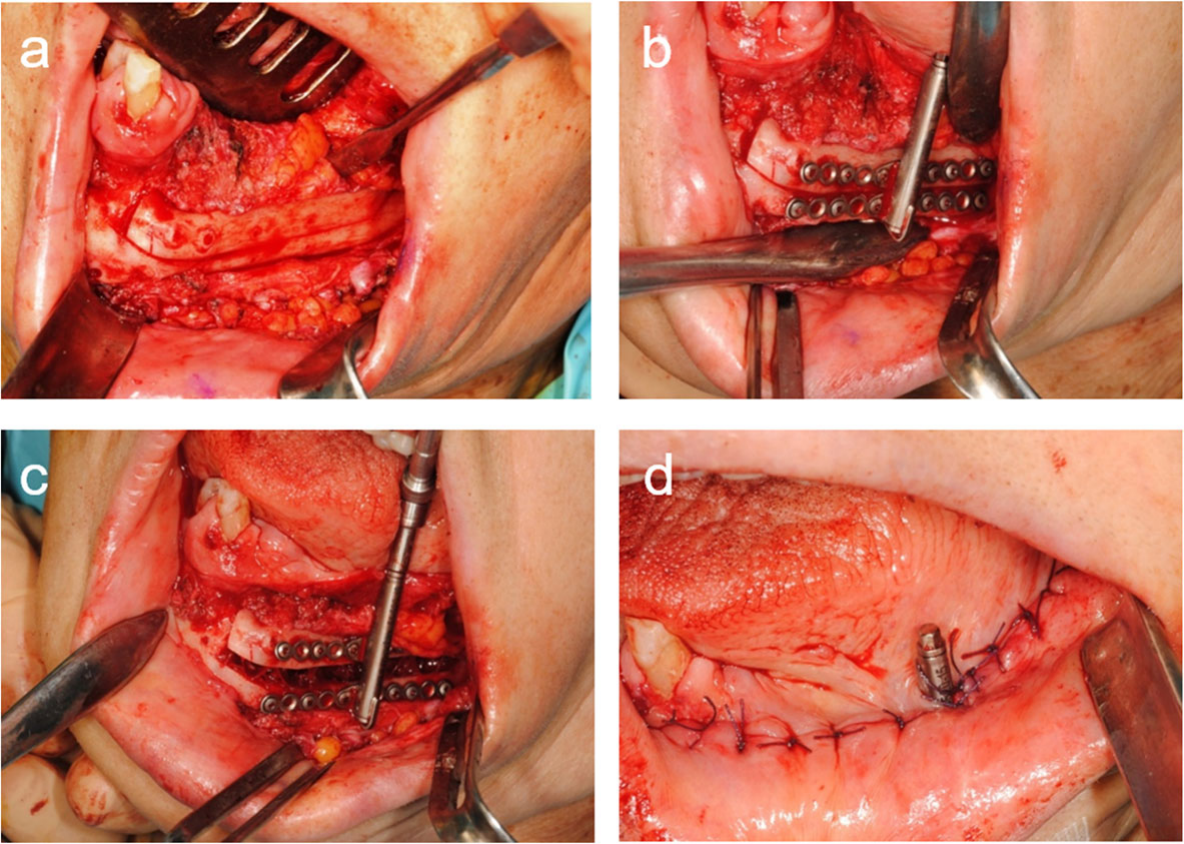
**a** Sagittal osteotomy was performed on the vestibular aspect of the fibula. **b** The distractor was fixed at the planned position. **c** The distractor was temporarily activated to a distance of approximately 5 mm to ensure correct function during distraction. **d** The osteotomized segment was repositioned at its initial position, and the wound was sutured

Vertical distraction osteogenesis of the fibula was carried out 1 year after reconstruction of the mandible. An intraoral incision was made in the buccal vestibule, and careful subperiosteal dissection was performed to obtain adequate visibility of the underlying bone, taking care to preserve the lingual mucoperiosteal attachment. As in case 1, an osteotomy was carried out after provisionally fixing the distraction device (TRACK 1.5-mm system, KLS Martin L.P.) and the device was re-fixed to confirm that it functioned as planned (Fig. 10).

**Fig. 11:**
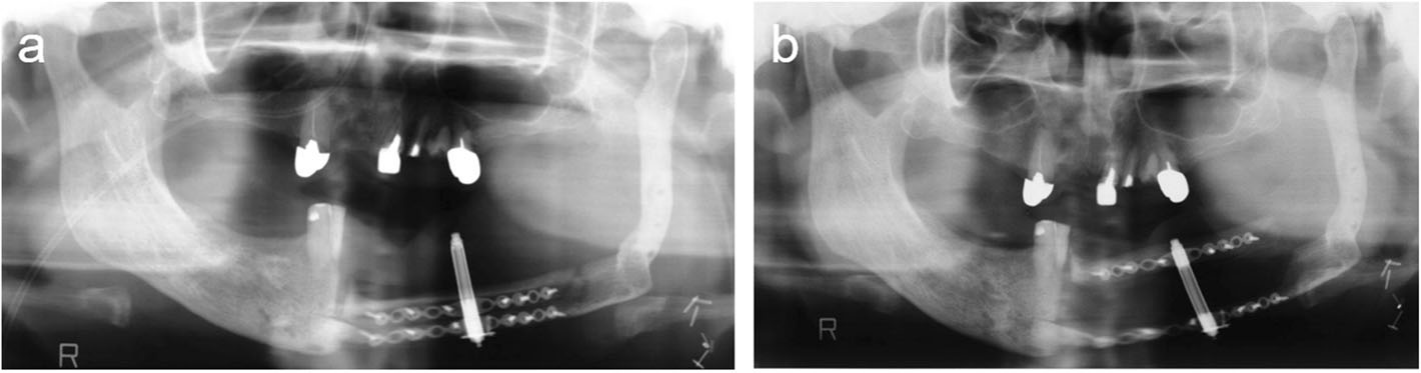
Orthopantomogram **a** 1 day postoperatively and **b** at the end of distraction osteogenesis

After a 6-day latency period, the distraction devices were activated at a rate of 1 mm/day by turning the device twice a day for 15 days. The bone was distracted by approximately 15 mm (Fig. 11).

**Fig. 12:**
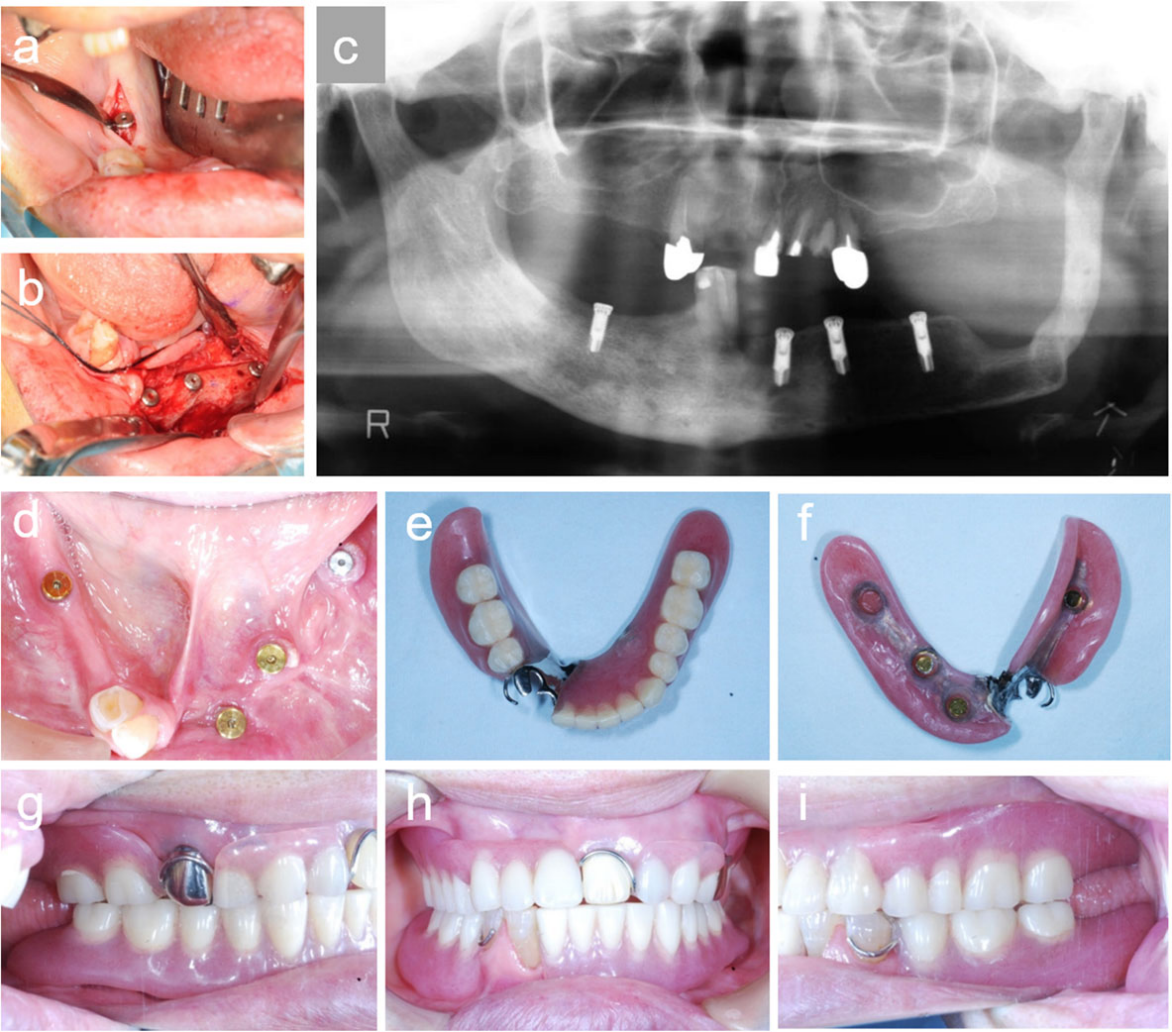
**a**, **b** Four dental implants were inserted into the distracted fibula. **c** Orthopantomogram after implantation. **d**–**i** Intraoral view after treatment: the implant denture was placed on the mandible

Osteogenesis was good 4 months after the end of vertical distraction, and an implant simulation was performed. The bone extender was removed, and four dental implants were implanted 6 months after the bone distraction. Finally, the implant denture was placed on the mandible (Fig. 12).

### Discussion

Tumor resection in the oral and maxillofacial region leads to facial deformity, stomatognathic system dysfunction, and subsequent psychological problems. Reconstruction of defects after tumor resection poses a common problem in oral and maxillofacial surgery [7, 8]. Free vascularized flaps are now considered safe and reliable for reconstruction of orofacial defects caused by tumor resection, and different donor sites such as the fibula, iliac crest, scapula, and radius have been suggested for use in reconstruction. The free vascularized fibula flap is widely used for the functional reconstruction of extended defects of both the mandible and the maxilla because it offers many advantages over other vascularized bone grafts for jaw reconstruction. The flap has sufficient length and better quality of the bone, which can be easily shaped with osteotomies, and the pedicle of the flap is of sufficient length for the reconstruction of the mandible or the maxilla [9–11]. However, the bone height of the fibula flap is insufficient. The fibula flap is also associated with some disadvantages with regard to desirable prosthetic rehabilitation with dental implants and the maintenance of adequate oral hygiene and negatively affects the profile of the reconstructed mandible [12]. The solutions to this problem may be the use of a double-barrel fibula flap graft [13–15] or secondary vertical distraction osteogenesis of the fibula flap [3, 16–19].

The double-barrel fibula flap graft technique was found to achieve greater bone height and to shorten the vertical distance to the occlusal plane, and the positive results of placement of dental implants in a double-barrel fibula flap were reported to achieve functional mandibular reconstruction [13–15]. However, it has been reported that the bridging of mandibular defects of > 9.0 cm in length is very challenging with the double-barrel technique due to the limited fibula length [13, 15].

Distraction osteogenesis is a technique used for the creation of neoformed bone by progressive stretching of bone segments obtained from a surgical osteotomy due to natural healing power, and this method offers an important advantage in that it is possible to extend the surrounding soft tissue together with bone distraction [19, 20]. Vertical distraction osteogenesis was initially used in cases of vertical defects of edentulous jaws to improve bone volume for dental implant placement [21], and it has become an effective technique to gain sufficient alveolar bone height in alveolar ridge atrophy. Histological results have demonstrated that distraction osteogenesis enables the formation of bone tissue of adequate quality and quantity, which could provide primary stability for implants [16]. Secondary vertical distraction osteogenesis of the fibula flap before implant therapy was reported to optimize the implant position and crown-to-implant ratio for ideal prosthetic rehabilitation [3, 16–19]. Some disadvantages of this technique include mispositioning of the distracted segment, bleeding in the osteotomy, painful tension, 'segment breakage and resorption during movements of transport segment, and the extended treatment period [22, 23]. In our cases, no major complications such as insufficient ossification, osteomyelitis, or relapse of the distraction were observed, although minor localized soft-tissue infections around the distraction rod during the waiting period of osteosclerosis were recognized and the bone formation in the extended area was somewhat poor. Therefore, careful observation should be performed during bone prolongation, and when infected findings are observed, antibiotics should be appropriately administered to control infection. Factors affecting bone formation after bone distraction include the age, waiting period, extension rate, extension frequency, extent of injury to the bone marrow and periosteum, blood supply to the bone fragments, infection, etc. Cancer patients are often elderly, and the moving bone fragments become smaller; therefore, the extension condition is not necessarily good.

In a comparative study of dental implant treatment outcomes following mandibular reconstruction with double-barrel fibula bone grafting or vertical distraction osteogenesis of the fibula, there was no significant difference in the rate of marginal bone loss between the two groups, although the incidence of the peri-implant inflammatory response in the double-barrel fibula bone grafting group was higher than that in the vertical distraction osteogenesis group [24]. Therefore, vertical distraction osteogenesis of the transplanted fibula is a suitable treatment option for performing optimal dental implant prosthesis by top-down treatment.

## Conclusions

Vertical distraction osteogenesis is a suitable treatment option for alveolar ridge deficiency resulting from fibula transplantation for mandibular reconstruction following tumor surgery.

## Abbreviations

CT: Computed tomography
EVPOME: Ex vivo-produced oral mucosa equivalent
